# General Versus Interventional Gastroenterologists: A Comparative Analysis of Follow-Up Outcomes After Endoscopic Mucosal Resection of Colorectal Polyps

**DOI:** 10.7759/cureus.76415

**Published:** 2024-12-26

**Authors:** Mahmoud Y Madi, Yassine Kilani, Hayden Rotramel, Michelle Baliss, Jill Elwing, Gregory Sayuk, Ahmad Bazarbashi

**Affiliations:** 1 Gastroenterology, Saint Louis University School of Medicine, St. Louis, USA; 2 Internal Medicine, Saint Louis University School of Medicine, St. Louis, USA; 3 Gastroenterology and Hepatology, Washington University School of Medicine, St. Louis, USA; 4 Medicine, Washington University School of Medicine, St. Louis, USA; 5 Gastroenterology and Hepatology, St. Louis Veterans Affairs Medical Center, St. Louis, USA; 6 Gastroenterology and Hepatology, Washington University in St. Louis, St. Louis, USA

**Keywords:** advanced endoscopy, colonoscopy and polypectomy, colon polyp removal, conventional endoscopic mucosal resection (cmer), gi endoscopy

## Abstract

Introduction

Colorectal cancer (CRC) represents a major global health burden, significantly impacting mortality rates and healthcare systems worldwide. CRC screening through colonoscopy enables early detection and removal of precancerous polyps. While standard polypectomy suffices for small polyps, larger ones require endoscopic mucosal resection (EMR). Though post-EMR surveillance is crucial for preventing recurrence, it remains unclear whether follow-up by general gastroenterologists yields comparable outcomes to surveillance by interventional specialists. This distinction carries significant implications for resource allocation, particularly given the limited availability of interventional gastroenterologists whose expertise is needed for other complex procedures. Our study examines this unexplored question by comparing post-EMR surveillance outcomes between these provider groups.

Methods

We conducted a retrospective study at the Saint Louis Veterans Affairs (VA) Health Care System of patients presenting for follow-up of colorectal polyp EMR between January 2019 and December 2022. Pre-defined variables extracted from the electronic medical record system were then analyzed to discern significant differences between general and interventional gastroenterologists’ outcomes. The primary outcome includes the rate of biopsy of scars after EMR between both groups. Additional outcomes include the number of polyps detected, detection of residual tissue at the EMR site, EMR site recurrence requiring polypectomy and mode of polypectomy, recommended surveillance interval suggested by the endoscopist, and the pathology of the EMR site biopsy.

Results

A total of 59 (N = 59) patients (median age: 67, mean age: 66.5 ± 6.6 years) met the inclusion criteria of our study. General gastroenterologists were more likely to biopsy the EMR site compared to interventional gastroenterologists (65% vs. 40%, p = 0.047). There was no difference in overall pathology detected when comparing general and interventional gastroenterologists (p = 0.074). While no EMR site biopsies were obtained in 16 patients (27.1%), there were no differences in the pathology of patients undergoing biopsy of the scar. Additionally, no significant differences were found in the Boston Bowel Preparation Score, number of polyps detected, detection of residual tissue at the EMR site, EMR site recurrence requiring polypectomy, or recommended surveillance interval.

Conclusion

Our study provides evidence that the outcomes of post-EMR follow-up are largely comparable between general and interventional gastroenterologists. Although general gastroenterologists exhibit higher rates of EMR site biopsy, the associated pathology shows no significant difference.

## Introduction

Colorectal cancer (CRC) remains a major oncological burden in the United States, ranking third in both incidence and cancer-related mortality among both genders [1]. While overall CRC incidence has exhibited a declining trend, there is a concerning increase in cases among individuals under 55 years old, constituting approximately 20% of new cases in 2019 [1]. This trend underscores persistent challenges in CRC prevention and emphasizes the necessity for ongoing vigilance and targeted interventions to reduce the risks of morbidity and mortality. Despite significant advances in screening methodologies, including stool tests, flexible sigmoidoscopy, and colonoscopy, there has been a concerning rise in the proportion of individuals diagnosed with advanced-stage CRC compared to the pre-screening era of the mid-1990s [1]. This highlights the challenges in early detection and stresses the need for continued efforts to promote timely screening and risk assessment.

Evidence indicates that resection of colorectal polyps results in a 60% reduction in CRC mortality [2]. Notably, larger adenomas pose a heightened risk of progression to invasive cancer, emphasizing the importance of adequate polyp resection [3]. Despite the rise of endoscopic submucosal dissection (ESD) in highly specialized academic medical centers, endoscopic mucosal resection (EMR) remains, in large part, the standard of care for the resection of large (≥20 mm) non-pedunculated colorectal polyps (LNPCPs) [4-6]. In the United States, EMRs are frequently performed by gastroenterologists with advanced endoscopy training or training in advanced tissue resection due to the inherent risk of perforation and bleeding associated with this procedure, the need for specialized training to manage potential complications, and the requirement of an endoscopy unit equipped with the personnel resources and tools needed for such a procedure [4,7]. Close follow-up post-EMR is essential to reduce the relatively high recurrence rates reported in the literature, which can range from 12% to 19% [8-10]. While published guidelines on EMR and large polyps are plentiful, there is currently no guidance to determine if endoscopic follow-up post-EMR should be completed by general gastroenterologists versus by advanced endoscopists performing the initial resection. While biopsy of post-EMR scar is recommended, practice patterns may vary amongst proceduralists [11]. At present, no literature outlining clinically significant differences in the outcomes of post-EMR follow-up when conducted by general gastroenterologists as opposed to advanced gastroenterologists exists.

Therefore, to further advance the current knowledge and potentially alleviate the workload on advanced gastroenterologists, we conducted a single-center retrospective study from January 2019 to December 2022 exploring differences in baseline characteristics and outcomes of patients undergoing post-EMR follow-up performed by gastroenterologists versus advanced endoscopists. An abstract version of this manuscript was previously presented as a meeting poster at the 2024 American College of Gastroenterology (ACG) Annual Meeting on October 27, 2024.

## Materials and methods

Data source

We conducted a retrospective study of individuals who received care at the Saint Louis Veterans Affairs (VA) Health Care System from January 2019 to December 2022. First, a search of electronic medical health records from the VA health care system was conducted to identify unique patient charts containing the American Medical Association Current Procedural Terminology (CPT) codes (45349, 45390, 44403), designating individuals who underwent colorectal polyp EMR. Second, we identified the subsequent visits for these patients using the VA healthcare system. This study was approved by the Associate Chief of Staff for the Research and Education Review Board at the VA Healthcare System as a quality improvement project.

Study population

Individuals aged 18 years or older who presented for follow-up of colorectal polyp EMR from 2019 to 2022 were included in the analysis. Medical records were retrospectively reviewed for a period of up to two years following the initial EMR procedure. A follow-up EMR was defined as an endoscopy unit visit in which a follow-up EMR was listed as a primary diagnosis. To capture this, we used CPT codes for EMR 45349, 45390, and 44403, in addition to retrospectively reviewing all the cases completed by the advanced endoscopists working at the VA center during the study period. We reviewed the procedural documentation to determine patients seen by gastroenterologists versus advanced endoscopists. The pathology of resected specimens at the initial EMR visit included tubular adenomas, tubulovillous adenomas, and sessile serrated polyps (with and without dysplasia). Patients with evidence of adenocarcinoma (including intramucosal or submucosal) were excluded. The study population did not include any non-English-speaking patients. None of the patients included had a history of CRC or inflammatory bowel disease.

Patient and hospital characteristics

We captured data available within the VA Health Care System on age, sex, race (White, African American), and comorbidities, including obesity, diabetes mellitus, coronary artery disease, antiplatelet or anticoagulant use, family history of CRC, and history of advanced adenoma. The endoscopists performing the procedures were divided into two groups: an advanced endoscopist pool consisting of two physicians and a general endoscopist pool consisting of six physicians. All the physicians included in the study had three or more years of experience in their respective fields.

Outcomes

Our primary outcome was the rate of endoscopist performance of EMR site biopsy and the biopsy results (e.g., normal, adenoma, dysplasia, malignancy) among patients undergoing post-EMR follow-up with gastroenterologists compared to advanced endoscopists. Secondary outcomes included the Boston Bowel Preparation Score, number of additional polyps detected, detection of residual tissue at the EMR site, EMR site recurrence requiring polypectomy and mode of polypectomy, recommended surveillance interval suggested by endoscopist, and the pathology of EMR site biopsy.

Statistical analysis

Categorical variables are presented as counts and percentages; continuous variables are presented as means with standard deviations and medians. Pearson’s chi-square, t-test, and Fisher's exact test were used to compare the outcomes among individuals undergoing EMR from gastroenterologists versus advanced endoscopists. We used IBM SPSS Statistics for Windows, Version 25 (Released 2017; IBM Corp., Armonk, New York, United States) for statistical analysis for this study.

## Results

Baseline characteristics

A total of 59 (N = 59) patients (median age: 67, mean age: 66.5 ± 6.6 years, 10% females, 16% African American) met the inclusion criteria of our study. When compared to individuals undergoing follow-up EMR with gastroenterologists, those following up with advanced endoscopists were older (median age: 68 vs. 66 years), with increased rates of obesity (21 (68%) vs. 12 (43%)), diabetes mellitus (15 (48%) vs. 12 (43%)), personal history of advanced adenoma (7 (26%) vs. 4 (14%)), and family history of CRC (2 (6.5%) vs. 1 (3.6%)), and decreased rates of CAD (7 (23%) vs. 10 (36%)) and antiplatelet or anticoagulant use (6 (19%) vs. 12 (43%)) (Table 1).

**Table 1:** Baseline characteristics of included patients GI: gastrointestinal physician; CRC: colorectal cancer

Characteristics	General GI (N = 28)	Interventional GI (N = 31)
Age (median, mean ± standard deviation)	66, 65.9 ± 7.1	68, 67.6 ± 6.2
Sex (N, percentage)		
Male	25 (89.3%)	28 (90.3%)
Female	3 (10.7%)	3 (9.7%)
Race (N, percentage)		
White	21 (75%)	26 (83.9%)
African American	7 (25%)	5 (16.1%)
Obesity (N, percentage)	12 (42.9%)	21 (67.7%)
Diabetes (N, percentage)	12 (42.9%)	15 (48.4%)
Coronary artery disease (N, percentage)	10 (35.7%)	7 (22.6%)
Antiplatelet or anticoagulant use (N, percentage)	12 (42.9%)	6 (19.4%)
Family history of CRC (N, percentage)	1 (3.6%)	2 (6.5%)

Biopsy results

**Figure 1:**
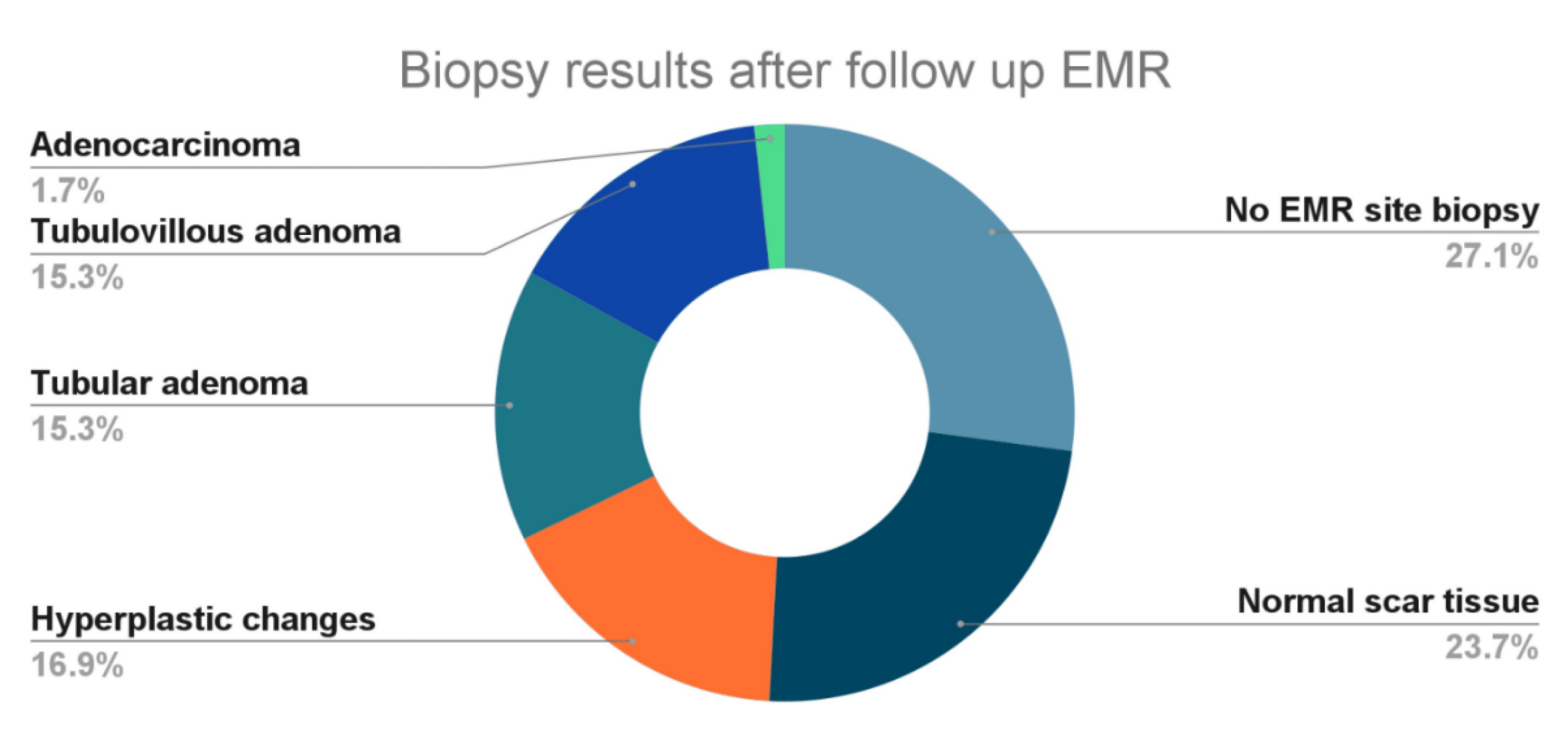
Biopsy results during first surveillance colonoscopy post-endoscopic mucosal resection of large colorectal polyps EMR: endoscopic mucosal resection

General gastroenterologists were more likely to biopsy the EMR site compared to interventional gastroenterologists (65% vs. 40%, OR 1.17, 95% CI 1.1-1.38, p = 0.047). No EMR site biopsies were obtained in 16 patients (27.1%). In the 43 patients undergoing biopsies of the scar, normal scar tissue was seen in 14 patients (23.7%), hyperplastic changes in 10 patients (16.9%), tubular adenoma in 9 patients (15.3%), tubulovillous adenoma in 9 patients (15.3%), and adenocarcinoma in 1 patient (1.7%) (Figure 1). There was no significant difference in overall pathology detected when comparing general and interventional gastroenterologists (p = 0.074).

Other outcomes

When comparing outcomes for follow-up colonoscopy after EMR, no significant differences were found in the Boston Bowel Preparation Score (p = 0.054), number of polyps detected (p = 0.081), detection of residual tissue at the EMR site (p = 0.384), EMR site recurrence requiring polypectomy (p = 0.383), or recommended surveillance interval (p = 0.8) (Table 2).

**Table 2:** Results of comparative analysis of general versus interventional gastroenterologists’ follow-up outcomes EMR: endoscopic mucosal resection Statistical tests used to yield p-values when appropriate included independent t-test, chi-square test, and Fisher's exact test.

Comparator	General gastroenterologists (N = 28)	Advanced endoscopists (N = 31)	Test statistic	p-value
Boston Bowel Preparation Score (median, mean ± standard deviation)	6.5, 6.4 ± 2.1	7, 6.5 ± 2.4	t: 0.17, df: 57	0.054
Tissue residual detection (number of events)	4	3	Fisher’s exact: 0.70	0.384
Recurrence requiring polypectomy (number of events)	5	3	Fisher’s exact: 0.72	0.383
Biopsy of EMR site (number of events)	29	14	Chi-square: 4	0.047
Pathology of EMR site biopsy	N/A	N/A	Chi-square: 2.1	0.074
Number of polyps detected (median, mean ± standard deviation)	3.5, 3.2 ± 1.8	3, 2.8 ± 2.2	t: 0.76, df: 57	0.081
Surveillance recommended (median, mean ± standard deviation)	12, 11.9 ± 3.1	10, 9.9 ± 2.3	t: 2.8, df: 57	0.8

## Discussion

Our study provides evidence that the outcomes of post-EMR follow-up are largely comparable between general and interventional gastroenterologists. Although general gastroenterologists exhibit higher rates of EMR site biopsy, the detected pathology was not significantly different. Furthermore, patients following with general gastroenterologists had similar Boston bowel preparation scores, number of polyps detected, detection of residual tissue at the EMR site, EMR site recurrence requiring polypectomy, and recommended surveillance interval.

EMR is a minimally invasive technique that facilitates faster resection of large colorectal polyps [12]. It is currently the standard of care for the resection of LNPCPs based on society guidelines [4-6]. Due to the higher-than-average risk of perforation and bleeding, EMRs are routinely performed by gastroenterologists trained in advanced endoscopy or advanced tissue resection in the United States [4,7]. Surveillance colonoscopies for polyps ≥20 mm resected in piecemeal fashion are recommended at approximately six months (first colonoscopy), one year (second colonoscopy) from the first, and three years (third colonoscopy) from the second surveillance [13]. This surveillance is essential to reduce the relatively high recurrence rates reported in the literature and is currently performed by both gastroenterologists and advanced endoscopists [9,10].

However, there is a gap in understanding whether there are significant differences in the endoscopic approaches and pathology outcomes of post-EMR surveillance colonoscopies when conducted by general gastroenterologists as opposed to interventional gastroenterologists. Our hypothesis is that outcomes are comparable between both groups, and this may alleviate procedural volume on advanced endoscopists who often see these patients for follow-up. To address this, we performed this single-center retrospective study, comparing the outcomes of individuals undergoing follow-up with advanced endoscopists as opposed to general gastroenterologists. Our results showed that although general gastroenterologists were more likely to biopsy the EMR site as compared to advanced gastroenterologists (65% vs. 40%, OR 1.17, p = 0.047), there were no significant differences in the biopsy results. Furthermore, patients followed by general gastroenterologists had similar Boston Bowel Preparation Scores, number of polyps detected, detection of residual tissue at the EMR site, EMR site recurrence requiring polypectomy, and recommended surveillance interval. These results suggest that follow-up colonoscopies post-EMR could be performed by general gastroenterologists in many cases, without notable differences in overall outcomes as compared to interventional endoscopists.

This study has several strengths. This is the first study that assesses the relationship between healthcare providers (general vs. advanced gastroenterologists) and the subsequent outcomes of the post-EMR follow-up colonoscopy, providing a unique set of data to help guide future efforts to replicate and verify these findings. Additionally, the ability to optimize resource utilization by possibly offloading the advanced endoscopists, particularly in centers where such services/resources may be limited and volume is superfluous, may be advantageous to patients. Furthermore, our analysis included a comprehensive overview of the outcomes of the post-EMR follow-up. This study also has some notable limitations. The single-center and retrospective nature of our study, coupled with our limited sample size to the veteran population, may limit the external validity and generalizability of our findings. However, addressing these biases can be achieved through further prospective, multicenter studies and meta-analyses. We anticipate that this trend will still be observed with much larger studies and potentially incorporated into future post-EMR guidelines.

## Conclusions

In conclusion, our study demonstrates that the outcomes of post-EMR follow-up colonoscopies are largely comparable between general gastroenterologists and interventional gastroenterologists. Although general gastroenterologists were more likely to biopsy the EMR site, no significant differences were found in pathology results or other key clinical outcomes, such as Boston Bowel Preparation Scores, polyp detection rates, detection of residual tissue, recurrence requiring polypectomy, and recommended surveillance intervals. These results suggest that general gastroenterologists can effectively manage post-EMR surveillance, potentially reducing the procedural load on advanced endoscopists, particularly in settings where resources are limited.

Our study provides a promising foundation for further research. Larger, prospective, multicenter studies are needed to confirm these results and potentially integrate them into future post-EMR guidelines. By optimizing resource utilization and expanding the role of general gastroenterologists in post-EMR care, this approach could enhance patient access to timely follow-up colonoscopies without compromising clinical outcomes.
